# Thiol-disulfide homeostasis and ischemia modified albumin levels in patients diagnosed with ovary carcinoma

**DOI:** 10.1038/s41598-025-04608-x

**Published:** 2025-06-05

**Authors:** Denizcan Hasturk, Selin Akturk Esen, Enes Seyda Sahiner, Ozcan Erel, Salim Neselioglu, Nurullah Aydogan, Muge Buyukaksoy, Ismet Seven, Sebnem Yucel, Dogan Uncu

**Affiliations:** 1 Clinic of Internal Medicine, Ankara Bilkent City Hospital, 06800 Ankara, Turkey; 2 Clinic of Medical Oncology, Ankara Bilkent City Hospital, 06800 Ankara, Turkey; 3 Clinic of Medical Biochemistry, Ankara Bilkent City Hospital, 06800 Ankara, Turkey

**Keywords:** Ovarian carcinoma, Oxidative stress, Thiol-disulphide, Total antioxidant capacity, Ischemia modified albumin, Biomarkers, Oncology

## Abstract

In our study, we determined the changes in the oxidative stress (OxS) biomarkers, thiol-disulfide (TD) homeostasis and ischemia-modified albumin (IMA) levels, in patients diagnosed with ovarian carcinoma before and after chemotherapy. We will examine the indirect effects of chemotherapy on OxS and antioxidant capacity by measuring changes in these blood biomarkers and compare the results with those of the healthy control group. This case–control study, which was conducted in a single-center, prospective design, included 42 patients diagnosed with ovarian cancer and 51 healthy volunteers. Venous blood samples were taken from all participants after 8 h of fasting, their serum was separated, and the serum total thiol, native thiol, disulfide, and IMA values were measured. In the comparison of the blood samples taken before the chemotherapy treatment of the patient group with the healthy control group, the native thiol (*p* < 0.001), total thiol (*p* < 0.001), and native thiol/total thiol (*p* < 0.001) values were found to be statistically significantly lower, and the disulfide (*p* = 0.001), disulfide/native (*p* < 0.001), and disulfide/total thiol (*p* < 0.001) levels were found to be statistically significantly greater. In the patient group diagnosed with ovarian cancer, a statistically significant difference was detected between the measurements of cancer antigen-125 (CA-125) (*p* < 0.001), native thiol (*p* = 0.019), and total thiol (*p* = 0.025) values at the 0th month before chemotherapy treatment and the third month after chemotherapy treatment. In the group that received adjuvant chemotherapy after the operation, the native thiol (*p* = 0.035), total thiol (*p* = 0.043), disulfide/native thiol (*p* = 0.035), disulfide total thiol (*p* = 0.035), native thiol/total thiol (*p* = 0.035) and IMA (*p* = 0.026) values were statistically significantly different between the diagnosis and third-month values. Our study suggests that TD homeostasis may be an important guide in terms of disease progression, complications during chemotherapy treatment, appropriate dose reductions, and modifications in chemotherapy depending on the toxicities experienced and the goals of the treatment.

## Introduction

Ovarian cancer was responsible for an estimated 314.000 new cases and 207.000 deaths globally in 2020^1^. It is the second most prevalent gynecological malignancy (after uterine cancer) and the leading cause of death among gynecological cancers^2^. Studies have demonstrated links between oxidative stress (OxS) and numerous diseases, including diabetes mellitus (DM), gliomas, leukemia, and ovarian cancer^3–6^. Understanding the balance of OxS plays a crucial role in the development, progression, and chemotherapy resistance of ovarian cancers^7–9^. OxS occurs when the balance between free radicals and antioxidants is disrupted^10–12^. Total antioxidant level measurement is one of the best methods used to evaluate the balance between free oxygen radicals and the antioxidant system.

Thiol-disulfide (TD) homeostasis and ischemia-modified albumin (IMA) levels have been confirmed as valuable biomarkers for OxS^13–15^. Thiols are important organic structures that play a vital role in antioxidant defense. They are oxidatively reduced by reactive oxygen species (ROS), forming disulfide bonds, which are vital indicators of the current OxS state^16^. This reversible process, termed TD homeostasis, is known to involve various mechanisms, such as antioxidant defense, detoxification, and apoptosis^16^. When the thiol levels decrease in serum, the antioxidant function also decreases. Therefore, increased disulfide levels are associated with increased OxS^17^. Additionally, the excessive generation of reactive oxygen radicals leads to IMA formation in vivo through the oxidative modification of serum albumin^18,19^. Hepatic and renal failure, acute infections, and carcinomas are associated with increased IMA levels, which are also elevated in numerous malignancies characterized by OxS imbalance^15^.

This study uniquely contributes new insights into the interplay between OxS and treatment response by providing a comprehensive analysis of TD homeostasis and IMA levels in ovarian cancer patients before and after chemotherapy. Our study aims to determine pre- and post-chemotherapy changes in TD balance, which forms a significant portion of total antioxidant levels in ovarian cancer patients, and IMA levels. This allows for an indirect examination of chemotherapy’s effect on OxS and antioxidant capacity. The results obtained will be compared with those of a healthy control group. With potential clinical implications for personalized ovarian cancer care, our study is novel in its examination of TD homeostasis and IMA levels in ovarian cancer patients as a potential guide for assessing disease progression, chemotherapy complications, and treatment modifications. There is limited data in the literature concerning between TD and IMA levels and chemotherapy.

## Materials and methods

### Study design

This single-center, prospective, observational study was conducted between June 2022 and June 2023 at the University of Health Sciences Ankara Bilkent City Hospital. The study included 42 patients with ovarian cancer who were followed up at the Medical Oncology outpatient clinics and 51 healthy volunteers who applied to the Internal Medicine outpatient clinics as the control group. Informed written consent was obtained from all participants.

### Population and participants

Patients aged 18 years and over with newly diagnosed ovarian cancer (with surface epithelial cell, germ cell, and sex cord-stromal tumor subtypes) at all stages (stage 1, 2, 3 and 4) were included in the study. The control group included healthy volunteers aged 18 years and over without chronic disease.

The exclusion criteria for volunteers were as follows: cognitive limitations; history of a second primary cancer; acute or chronic disease associated with ischemia; use of vitamin A, vitamin C, or vitamin E supplements; history of rheumatic disease; active infection; use of immunosuppressive or anti-inflammatory drugs; history of cardiovascular disease, diabetes, or liver, kidney, or thyroid dysfunction; smoking; alcohol consumption; and inability to feed orally.

### Data collection

After the systemic examinations of all the volunteers who participated in the study, age was recorded during the face-to-face interviews; histopathological diagnoses, laboratory values, and treatments applied to the patients with ovarian cancer were obtained from the hospital information management system.

### Laboratory analysis

Blood samples were collected from patients with ovarian cancer before the first chemotherapy and surgery, and after the third month of chemotherapy. Blood samples from the healthy volunteer group were collected from blood samples requested for any other test during routine check-up visits to the internal medicine polyclinic. After 8 h of fasting, 5 ml of blood samples were taken from the volunteers, allowed to stand for 20 min, and centrifuged at 1500 rpm for 10 min. The plasma and serum were then separated. The separated serum samples were stored at -80 °C. Plasma TD homeostasis was determined according to the method described by Erel and Neselioğlu^16^. In this method, dynamic disulfide bonds (–S–S) are reduced to functional thiol groups (sulfhydryl groups) with sodium borohydride (NaBH4)^16^. Unused NaBH4 is removed with formaldehyde, while the total thiol amount is calculated via spectrophotometric measurement of the chromogenic compound formed with the modified Elmann reagent at a wavelength of 415 nm. The disulfide value was determined by subtracting the native thiol value from the total thiol value and dividing the result by two. After determining the native thiol, total thiol, disulfide levels, disulfide/native thiol, disulfide/total thiol, and native thiol/total thiol ratios were calculated and determined as percentages according to a previously described procedure^16^. Serum IMA levels were measured as follows: 50 μL of 0.1% cobalt chloride was added to the plasma samples (200 μL), and the mixed solution was incubated for 10 min. 50 μL of 1.5 mg/ml dithiothreitol was added as a colorimetric agent^20^. The binding reaction was stopped after 2 min by adding 1 ml of 0.9% NaCl. The absorbances of the samples were then measured at 470 nm with a spectrophotometer. Additionally, the color of the samples containing dithiothreitol was compared with the color of the colorimetric control tubes. The obtained results were expressed as absorbance units (ABSU).

### Survival analysis

Patient survival information was obtained from the National Population Registration and Tracking System database. Progression-free survival (PFS) was defined as the time from the initiation of first-line chemotherapy to progression or death from any cause. Overall survival (OS) was defined as the time from disease onset to death from any cause or the last follow-up.

### Ethical approval

The study was approved by the Ankara Bilkent City Hospital Ethics Committee of the Ministry of Health of the Republic of Turkey, decision number E1-22–2616, according to good clinical practice, and applicable laws, and the Declaration of Helsinki.

### Statistical analyses

Statistical analyses were performed via IBM SPSS Statistics for Windows Version 25.0 (Statistical Package for the Social Sciences, IBM Corp., Armonk, NY, USA). Descriptive statistics are presented as frequencies and percentages for categorical variables, and mean ± SDs, medians (IQRs) for continuous variables. When the study data were examined in terms of normality assumptions, Kolmogorov–Smirnov values were determined as p < 0.05. Therefore, the Kruskal Wallis H test was applied to determine whether there was a significant difference between the continuous variables and the Conut score variable groups. The Bonferroni-corrected Mann–Whitney U test, which was used for subgroup comparisons. Categorical variables were compared via the chi-square test. Statistical significance was set at *P* < 0.05 significance.

## Results

The study included 93 participants, 42 of whom were diagnosed with ovarian carcinoma and 51 of whom were in the healthy control group. The mean age at diagnosis in the patient group was 59.88 years, and the mean age in the control group was 58.67 years (*p* = 0.07). The mean tumor size in the patient group was 89.2 mm. The clinical and demographic characteristics of the volunteers included in the study are summarized in Table 1.

**Table 1:** Clinical and pathological characteristics of the patients.

Variables	Mean ± SD	Median (IQR)	
Diagnosis Age (years)	59.88 ± 14.25	57.50 (22.25)	
Tumor size (mm)	89.20 ± 48.21	80.00 (67.00)	

Variables	Group	Frequency	Percentage (%)
Family history of ovarian cancer	No	40	95.2
	Yes	2	4.8
Complaint	Abdominal pain	17	40.5
	Abdominal distension	8	19.0
	Abdominal mass	7	16.7
	Vaginal bleeding	5	11.9
	Dyspnea	1	2.4
	Abdominal pain and distension	4	9.5
ECOG-PS	0	3	7.1
	1	26	61.9
	2	11	26.2
	3	2	4.8
Histopathological diagnosis	Surface epithelial	39	92.9
	Germ cell	1	2.4
	Sex cord stroma	2	4.8
Ovarian cancer subtypes	Serous	35	83.3
	Endometrioid	2	4.8
	Clear cell	2	4.8
	Mixed histology	1	2.4
	Granulosa cell tumor	2	4.8
Surface epithelial	Low grade	2	5.1
	High grade	37	94.9
First-line treatment	Surgery + Adjuvant Chemotherapy	18	42.9
	Neoadjuvant Chemotherapy + Surgery + Adjuvant Chemotherapy	15	35.7
	Metastatic first-line chemotherapy	9	21.4
Pathological stage	1	0	0
	2	4	9.5
	3	19	45.2
	4	19	45.2
Status	Dead	6	14.3
	Alive	36	85.7

SD: Standard deviation; ECOG PS: Eastern Cooperative Oncology Group performance status

As shown in Table 2, there was a statistically significant difference in uric acid (*p* = 0.001), native thiol (*p* < 0.001), total thiol (*p* < 0.001), disulfide (*p* = 0.001), disulfide/native thiol (*p* < 0.001), disulfide/total thiol (*p* < 0.001), and native thiol/total thiol (*p* < 0.001) values between the control group and the patient group who did not receive chemotherapy at the 0th month. The uric acid, disulfide, disulfide/native thiol, and disulfide/total thiol values were higher in the patient group than in the control group. The native thiol, serum total thiol, and native thiol/total thiol values were lower in the patient group than in the control group.

**Table 2:** Comparison of TD Balance and IMA Levels of Healthy Controls and Patients at Diagnosis.

	Variables	Patient group n = 42 (0th month) Median (IQR)	Control group n = 51 Median (IQR)		*P*-value
Diagnosis	Total Protein (g/dL)	71.50 (8.00)	70.00 (5.00)		0.503
	Albumin (g/dL)	43.50 (8.00)	44.00 (4.00)		0.500
	Uric Acid (mg/dL)	5.00 (2.40)	4.20 (1.20)		**0.001**
	NT (μmol/L)	458.95 (101.42)	513.10 (122.90)		** < 0.001**
	TT(μmol/L)	497.38 (94.47)	544.12 (94.46)		** < 0.001**
	DS (μmol/L)	20.64 (6.99)	15.95 (9.49)		**0.001**
	DS/NT (%)	4.88 (2.52)	3.20 (2.33)		** < 0.001**
	DS/TT (%)	4.44 (2.08)	3.01 (2.06)		** < 0.001**
	NT/TT (%)	91.11 (4.20)	93.99 (4.12)		** < 0.001**
	IMA (ABSU)	0.70 (0.04)	0.70 (0.03)		0.284

Mann Whitney U test, p-value of less than 0.05 was considered statistically significant given in bold.

n: Patient number; TD: Thiol-disulfide; NT: Native thiol; TT: Serum total thiol; DS: Disulfide; DS/NT: Disulfide/native thiol; DS/TT: Disulfide/total thiol; NT/TT: Native thiol/total thiol; IMA: Ischemia modified albumin.

As shown in Table 3, statistically significant differences were found between the pre-chemotherapy and post-chemotherapy measurements of total protein (*p* = 0.031), albumin (*p* = 0.035), Lactate dehydrogenase (LDH) (*p* = 0.002), Cancer antigen-125 (Ca125) (*p* < 0.001), native thiol (*p* = 0.019), and serum total thiol (*p* = 0.025) levels. The total protein, albumin, LDH, Ca125, native thiol, and serum total thiol values were lower after chemotherapy than before.

**Table 3:** Comparison of laboratory values, TD balance, and IMA levels in the patient group before (0th month) and after chemotherapy (3rd month).

Variables	Pre-chemotherapy (0th month) Median (IQR)	Post-chemotherapy (3rd month) Median (IQR)	*P*-value
Total protein (g/L)	71.50 (8.00)	69.00 (7.50)	**0.031**
Albumin (g/dL)	43.50 (8.00)	42.00 (5.00)	**0.035**
Uric acid (mg/dL)	5.00 (2.38)	4.60 (1.40)	0.057
LDH (U/L)	232.00 (118.50)	197.00 (65.50)	**0.002**
Ca 125 (U/mL)	223.50 (1057.03)	11.90 (41.55)	** < 0.001**
CEA (ng/mL)	0.59 (1.53)	0.78 (1.17)	0.144
AFP (µg/L)	1.90 (1.45)	1.40 (2.30)	0.060
bHCG (mIU/mL)	0.00 (3.25)	0.00 (3.25)	0.403
NT (μmol/L)	458.95 (101.43)	417.70 (90.75)	**0.019**
TT (μmol/L)	497.39 (94.48)	463.01 (77.81)	**0.025**
DS (μmol/L)	20.65 (7.00)	21.16 (7.16)	0.221
DS/NT (%)	4.88 (2.52)	5.11 (2.70)	0.060
DS/TT (%)	4.45 (2.08)	4.63 (2.21)	0.062
NT/ TT (%)	91.11 (4.20)	90.56 (4.42)	0.053
IMA (ABSU)	0.70 (0.04)	0.69 (0.04)	0.212

Wilcoxon test, the p-value of less than 0.05 was considered statistically significant given in bold.

LDH: Lactate dehydrogenase; CA 125: Cancer antigen-125; CEA: Carcinoembryonic antigen; AFP: Alpha-fetoprotein; bHCG: Beta-human chorionic gonadotropin; TD: Thiol-disulfide; NT: Native thiol; TT: Serum total thiol; DS: Disulfide; DS/NT: Disulfide/native thiol; DS/TT: Disulfide/total thiol; NT/TT: Native thiol/total thiol; IMA: Ischemia modified albumin.

There was a statistically significant difference between the values of native thiol (*p* = 0.035), serum total thiol (*p* = 0.043), disulfide/native thiol (*p* = 0.035), disulfide/total thiol (*p* = 0.035), native thiol/total thiol (*p* = 0.035), and IMA (*p* = 0.026) before surgery (before neoadjuvant chemotherapy) and after neoadjuvant chemotherapy in the surgery + adjuvant chemotherapy group. The native thiol, serum total thiol, native thiol/total thiol, and IMA values after neoadjuvant chemotherapy were lower than those before surgery (before neoadjuvant chemotherapy); the disulfide/native thiol and disulfide/total thiol values after neoadjuvant chemotherapy were higher than those before surgery (before neoadjuvant chemotherapy). There was no statistically significant difference between the disulfide (*p* = 0.170) values before and after neoadjuvant chemotherapy (Table 4).

**Table 4:** Comparison of TD balance and IMA levels before (0th month) and after (3rd month) chemotherapy according to the first treatment modality applied in the patient group.

Treatment	Variables	Before chemotherapy (0th month) Median (IQR)	After chemotherapy (3rd month) Median (IQR)	*P*-value
Surgery + Adjuvant Chemotherapy (n = 18)	NT (μmol/L)	468.30 (77.93)	455.55 (81.62)	**0.035**
	TT (μmol/L)	502.27 (73.60)	488.79 (77.28)	**0.043**
	DS (μmol/L)	19.52 (7.57)	19.22 (8.03)	0.170
	DS/NT (%)	4.02 (2.83)	4.54 (2.04)	**0.035**
	DS/TT (%)	3.72 (1.96)	4.17 (1.70)	**0.035**
	NT/TT (%)	92.56 (4.55)	91.68 (3.41)	**0.035**
	IMA (ABSU)	0.70 (0.04)	0.68 (0.02)	**0.026**
Neoadjuvant Chemotherapy + Surgery + Adjuvant Chemotherapy (n = 15)	NT (μmol/L)	414.20 (111.50)	401.10 (114.23)	0.331
	TT (μmol/L)	459.51 (109.36)	449.88 (118.27)	0.272
	DS (μmol/L)	20.68 (5.66)	20.41 (5.93)	0.638
	DS/NT (%)	5.47 (3.04)	4.79 (2.53)	0.551
	DS/TT (%)	4.93 (2.45)	4.37 (2.09)	0.551
	NT/TT (%)	90.14 (4.90)	90.84 (4.16)	0.594
	IMA (ABSU)	0.70 (0.06)	0.70 (0.06)	0.755
Chemotherapy in Inoperable/Metastatic Disease (n = 9)	NT (μmol/L)	465.50 (123.10)	390.40 (90.50)	**0.011**
	TT (μmol/L)	496.63 (105.90)	438.45 (82.76)	**0.015**
	DS (μmol/L)	21.77 (9.10)	24.02 (4.47)	0.086
	DS/NT (%)	4.84 (3.37)	6.15 (2.73)	**0.038**
	DS/TT (%)	4.41 (2.78)	5.48 (2.12)	**0.033**
	NT/TT (%)	91.18 (5.56)	89.05 (4.22)	**0.028**
	IMA (ABSU)	0.70 (0.05)	0.69 (0.07)	0.280

Wilcoxon test, *p*-value of less than 0.05 was considered statistically significant given in bold.

n: Patient number; TD: Thiol-disulfide; NT: Native thiol; TT: Serum total thiol; DS: Disulfide; DS/NT: Disulfide/native thiol; DS/TT: Disulfide/total thiol; NT/TT: Native thiol/total thiol; IMA: Ischemia modified albumin.

There was no statistically significant difference between the values of any variables before and after neoadjuvant chemotherapy (before surgery) in the group that received neoadjuvant chemotherapy and underwent surgery (Table 4).

In the inoperable/metastatic first-line chemotherapy group, there was a statistically significant difference in the native thiol (*p* = 0.011), serum total thiol (*p* = 0.015), disulfide/native thiol (*p* = 0.038), disulfide/total thiol (*p* = 0.033), and native thiol/total thiol (*p* = 0.028) values before chemotherapy and at the third month of chemotherapy. The values of native thiol, serum total thiol, and native thiol/total thiol at the third month of chemotherapy were lower than those before chemotherapy; the values of disulfide/native thiol and disulfide/total thiol at the third month of chemotherapy were greater than those before chemotherapy. There was no statistically significant difference between the values of disulfide (*p* = 0.086) and IMA (*p* = 0.280) at the third month of chemotherapy and the values before chemotherapy (Table 4).

**Fig. 1:**
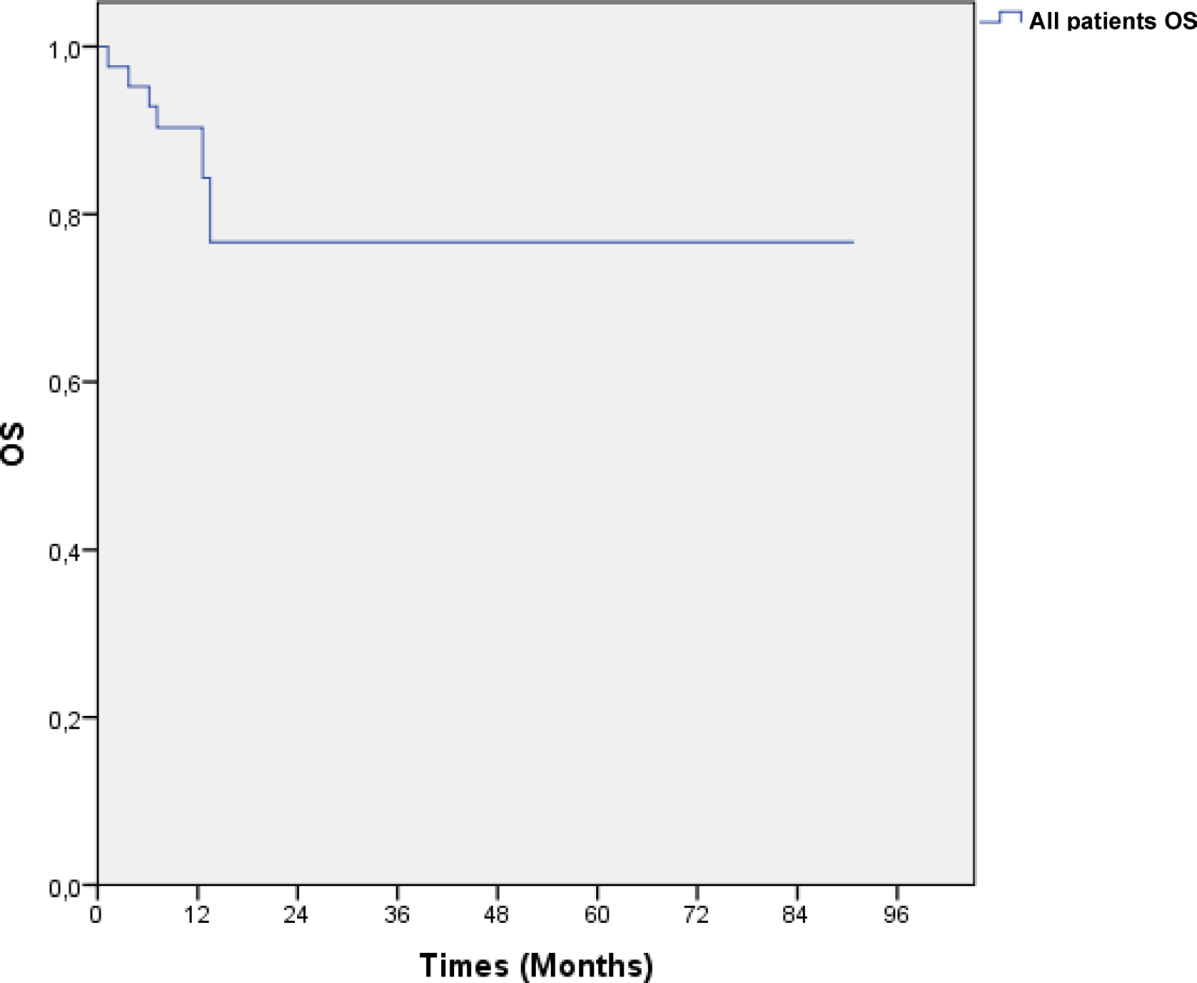
Kaplan–Meier curve showing the OS of the entire patient group. *OS: Overall survival.*

As seen in Fig. 1, the patients’ overall survival (OS) did not reach the median in the Kaplan–Meier survival analysis. The 2-year OS was determined to be 90%.

## Discussion

ROS contribute to cancer pathogenesis, which is also supported by recent studies^21,22^. In cancer treatment, the main goal is to prevent the proliferation of cancer cells and the OxS that triggers the pathophysiological mechanisms that cause these cells to form and proliferate. The biochemical measurement of OxS levels may be used to evaluate different cancer treatment options and predict the course of the disease.

In our study, statistical analysis of the blood samples taken from the patient at the time of diagnosis revealed that the native thiol, serum total thiol, and native thiol/total thiol values were statistically significantly lower than those of the healthy control group. In contrast, the disulfide, disulfide/native thiol, and disulfide/total thiol levels were statistically significantly higher. In different studies evaluating different cancer types, it has been observed that total thiol and native thiol levels were lower in prostate, breast, lung, and endometrial cancer patients than in the control group patients^23–26^. In our study, results consistent with these studies were obtained in terms of total thiol and native thiol levels. Similar to our study, Sezgin B. et al. also reported elevated disulfide, disulfide/native thiol, and disulfide/total thiol levels in a patient group compared with a control group in their assessment of TD homeostasis in cervical cancer patients^27^. However, in contrast to our study, according to the study by Erel and Neselioğlu, disulfide levels were lower in patients with malignant proliferative tumors of the colon and kidney than in healthy controls^16^. The different results obtained in our study may be due to the different pathophysiological mechanisms of solid malignancies. In a study by Aktürk Esen and colleagues on autosomal dominant polycystic kidney disease, serum native thiol and total thiol levels were lower in the patient group than in the healthy group, which is consistent with our study^28^. These results suggest that determination of thiol balance may be an important biomarker to measure antioxidant capacity, although the pathophysiological process was completely different from our patient group.

In the patient group with ovarian cancer, a statistically significant difference was found between the native thiol and total thiol values between the diagnosis and third-month measurements, and the measurements at the third month were observed to be lower than the measurements at time of diagnosis. These results may suggest that the antioxidant capacity of ovarian cancer patients decreases significantly with chemotherapy.

In the group receiving adjuvant chemotherapy after surgery, the values of the native thiol, serum total thiol, native thiol/total thiol, and IMA variables at the third month of chemotherapy were lower than those before chemotherapy; the values of the disulfide/native thiol and disulfide/total thiol variables at the third month of chemotherapy were greater than those before chemotherapy. As mentioned, these findings suggest that antioxidant capacity may decrease and that OxS may increase after chemotherapy. This situation strengthens the possibility that chemotherapy-related side effects may increase in the later stages of chemotherapy. In a study by Topuz et al. on the relationship between adjuvant chemotherapy and TD in patients with breast cancer and lymphoma, it was found that thiol levels were low, and disulfide and disulfide/total thiol levels were high in the group with chemotherapy-related cardiotoxicity^29^. The balance of antioxidant and OxS is thought to be disrupted, and the number of chemotherapy side effects increases as the number of chemotherapy cycles increases. To clarify this hypothesis, studies investigating the relationships among antioxidant capacity, OxS, and the side effects of chemotherapy are needed.

In the group that underwent surgery after neoadjuvant chemotherapy, there was no statistically significant difference in any of the variables between the values at the third month of chemotherapy and the values before chemotherapy in any of the variables. The fact that TD balance and IMA levels did not differ in the values of the patients before and after neoadjuvant chemotherapy suggests that the decreased tumor burden may reduce OxS, but the chemotherapy administered may reverse this situation, so OxS and total antioxidant capacity may remain in balance. In this study, in the group that had inoperable/metastatic ovarian cancer and received first-line chemotherapy, the values of the native thiol, total thiol, and native thiol/total thiol variables at the third month of chemotherapy were lower than those before chemotherapy; the values of the disulfide/native thiol and disulfide/total thiol variables at the third month of chemotherapy were greater than those before chemotherapy. There was no statistically significant difference between the disulfide and IMA values at the third month of chemotherapy and before chemotherapy. We thought that this situation might be related to the deterioration of oxidative balance and increased OxS after chemotherapy. Similarly, in a study by Akyol et al. on the relationship between TD and chemotherapy in breast, ovarian, and endometrial cancer patients, high disulfide/native thiol and disulfide/total thiol ratios were associated with increased OxS after chemotherapy^30^. We hypothesize that the observed differences in outcomes between the neoadjuvant chemotherapy and inoperable/metastatic ovarian cancer groups could be associated with the effects of tumor load and disease stage on the OxS balance.

In our study, we measured TD and IMA levels, which have been used as OxS biomarkers in various disease groups in the literature. We specifically investigated the relationship between these values and chemotherapy treatment applied to ovarian cancer patients. Given that antineoplastic agents such as platinum group drugs, and taxanes are known to accelerate tumor apoptosis, elevate OxS, leading to ROS-induced damage and reduced antioxidant capacity. Moreover, intravenous steroids (dexamethasone) used as premedication may have further contributed to increased OxS^31–34^.

Our study had several limitations. Owing to its prospective nature, the follow-up period was short, and overall survival did not reach the median. An expanded follow-up period, such as including a 6-month time point, would enable a more comprehensive assessment of the utility of these biomarkers in monitoring treatment response and long-term side effects. Since it included a small number of heterogeneous patient groups (nonmetastatic, metastatic, and different histological subtypes), performing subgroup analyses according to disease stage was difficult. Furthermore, the variation in chemotherapy regimens among patients made it difficult to achieve a consistent assessment across the study population. In our study, due to the small patient group and the fact that OS did not reach the median, we could not evaluate the prognostic factors affecting survival of patients and the comparative analysis of subgroups. Another major limitation of our study is that we were not able to evaluate chemotherapy side effects according to CTCAE version 5.0. The lack of side effect evaluation suggests the power of the study. Additionally, there were some differences in demographic characteristics among the patients. However, due to insufficient data on the impact of age and family history of cancer on TD and IMA levels between the groups, it is unclear whether these differences influenced the results.

In addition to these limitations, the study also has strengths. Importantly, this is the first study in the literature to examine TD and IMA homeostasis before and after chemotherapy in patients with ovarian cancer. Considering the effect of OxS on the treatment process of ovarian cancer patients, we believe that treatment and follow-up plans should be planned according to these biomarkers and that TD and IMA biomarkers can be easily accessible, and that they can be pioneers for patient-specific treatments in future studies with treatment and follow-up evaluations. While studies on patient-specific and targeted treatments continue, we anticipate that they can shed light on other studies in the literature.

## Conclusions

In this study, TD balance, which is formed by thiols and constitutes a significant part of the total antioxidant level, and disulfide levels, which increase with OxS, were disrupted in patients with ovarian carcinoma compared to the healthy control group. This suggests that increased OxS and decreased total antioxidant capacity may be effective in the formation of ovarian cancer, while it suggests that OxS may have increased and total antioxidant capacity may have decreased after cancer formation. In addition, when evaluated before and after chemotherapy, it suggests that antioxidant capacity may have decreased and OxS may have increased after chemotherapy in ovarian cancer patients. This strengthens the possibility that chemotherapy-related side effects may increase in the later stages of chemotherapy. In our opinion, TD homeostasis may be an important guide for appropriate dose reductions and modifications in chemotherapy depending on complications, toxicities and treatment goals during chemotherapy treatment. In light of new studies investigating OxS and antioxidant response in patients with ovarian carcinoma, patient-specific targeted therapies targeting the oxidant-antioxidant balance can be developed to achieve better survival rates. These hypotheses can be elucidated more clearly with larger-scale studies involving participants from different institutions and different geographies.

## Data Availability

Raw data supporting the conclusions of this study will be made available by the author upon request. Data requests can be made to the corresponding author via this email: denizcanh@hotmail.com.
